# Association of violence against women with religion and culture in Erbil Iraq: a cross-sectional study

**DOI:** 10.1186/1471-2458-12-800

**Published:** 2012-09-17

**Authors:** Namir Ghanim Al-Tawil

**Affiliations:** 1 Department of Community Medicine, College of Medicine, Hawler Medical University, Erbil, Iraq

**Keywords:** VAW (Violence Against Women), Culture, Religion, Intimate partner’s violence

## Abstract

**Background:**

Violence against women by intimate partners is still a public health problem. The study aims at finding out the prevalence of violence among women residing in Erbil city (Muslim culture) and in Ankawa sub-district (Christian culture), finding out the role of religion and culture on the prevalence, and finding out some other factors (like occupation of husband and wife, age at marriage, woman agreement for marriage, illegitimate relations of husband) that might be associated with violence.

**Methods:**

A cross-sectional study was carried out in Erbil during the second half of the year 2011. Two groups were considered; group one (G1) included women residing in Ankawa sub-district (representing Christian culture), and group two (G2) included women residing in Erbil city (representing Muslim culture). A convenience method of sampling was used to collect the sample (250 women of each group). Questionnaire was designed to collect information about history of exposure to physical, sexual, and psychological violence, in addition to the related factors. These forms were distributed (by women of the *Assyrian Women Union)* in sealed envelopes to women attending the Mass in three churches located in Ankawa. Women of Erbil group were recruited from the maternity teaching hospital of Erbil. The same questionnaire was distributed to them by the same team. Binary logistic regression was used to show the independent effect of each factor on the prevalence of violence.

**Results:**

Overall prevalence of violence (physical and/or sexual) in G2 (*20.8%*) was higher than that of G1 (*18.8%*). The prevalence of psychological violence was 40% in Erbil, which was significantly higher than the prevalence (24.8%) of Ankawa. The rates of physical and sexual violence were also higher in Erbil (18.4%, and 10.8% respectively) than rates of Ankawa (16.8% and 8% respectively). Factors found to be significantly associated with overall violence were: culture of Erbil, alcoholic husband, wife working as manual worker (compared with professionals), and having children.

**Conclusion:**

Violence against women is a serious public health issue. There was significant role of culture on the prevalence of violence.

## Background

The United Nations defines violence against women as any act of gender-based violence that results in, or is likely to result in, physical, sexual or mental harm or suffering to women, including threats of such acts, coercion or arbitrary deprivation of liberty, whether occurring in public or in private life [1].

The WHO comprehensive study on domestic violence (DV) revealed that intimate partner violence (IPV) is the most common form of violence among women, much more than assault or rape by strangers [2]. Abused women by intimate partners have 50–70% increasing incidence of gynecological, central nervous system, and stress related problems [3]. Nearly 50 population-based surveys from 35 nations around the world find that from 10 to over 50% of women are physically assaulted by intimate partners during their lifetimes [4].

In Baghdad, Iraq, a study done in 2006 showed that the percentage of DV committed by husbands was 57.6% for a one time incidence during life time [physical violence (39.3%), sexual violence (14.9%), and psychological violence (57.3%)], and 44% for current violence [5].

A recent study conducted in Erbil on 800 women showed that the *past year* prevalence of any form of violence was 45.3%; while it was 43.3% for psychological violence, 15.1% for physical violence, and 12.1% for sexual violence [6].

The current study is going to compare violence rate between two groups of women, the first one living in Ankawa sub-district of Erbil, and are Christians, whereas the second group of women are living in Erbil city center, and are Muslims. Up to the researcher’s knowledge, no previous study had been conducted to find out the prevalence of violence in Ankawa sub-district.

The study aims at finding out the prevalence of violence (overall, psychological, physical, and sexual) among both groups of women, finding out the role of religion and culture on the prevalence, and finding out some other factors that might be associated with violence.

## Methods

A cross-sectional study was carried out in Erbil during the second half of the year 2011. Erbil city is the main city of Erbil governorate which is the capital of Kurdistan region, located North of Iraq. Erbil city mostly inhabited by Kurdish people whose religion is Muslim. Several decades ago, Ankawa was a sub-district located near to Erbil city, and inhabited by Christian people who have different cultural values of people living in Erbil. Now, Ankawa is considered as part of Erbil city owing to the expansion of Erbil. Two groups of women were considered; group one (G1) included women residing in Ankawa sub-district, and group two (G2) included women residing in Erbil city. A convenience method of sampling was used to collect the sample (250 women of each group). Inclusion criteria for G1 were married, Christian women, living in Ankawa sub-district; while for G2, inclusion criteria were married, Muslim, Kurdish women residing in Erbil city. At least one year of marriage is needed for the woman (of both groups) to be included in the study. A questionnaire adapted from WHO multi country study was used for data collection [2]. Modifications were carried out as the controlling behaviors were not considered, and the life time violence was not investigated. It included questions about the socio-demographic characteristics of women, also questions related to psychological, physical, and sexual types of violence. Psychological violence was defined as presence of any of the following acts during past year: insulting, scaring/intimidation, humiliation, threatening to remarry, and threatening to harm. Physical violence was defined as presence of any of the following acts during the past year: slapping on face, pushing/shaking/throwing something, twisting arm/pulling hair, hitting with fist/something, and kicking/dragging. Lastly, the sexual violence was defined as physically forcing the woman to have sexual intercourse, or forcing her to perform any sexual act. The overall (period) prevalence of violence was defined as having history of physical and/or sexual violence during the past year. Occupations of women and their husbands were categorized into four categories: the first was the high professionals like doctors, dentists, pharmacists, engineers, lawyers, in addition to managers and business owners. The second category was the non-manual skilled, like teachers, clerks, nurses, policemen and military personnel. The third one was the manual, skilled or unskilled, workers including farmers and drivers as examples. The last category was the unemployed. Alcohol drinking by husband was categorized into three categories: daily drinking, two to three times per week, and on occasions.

Verbal consent was taken from women after briefing them with the aims of the study, and ensuring the confidentiality of the study. Approval was taken from the research Ethics committee of the college of medicine, in addition to the official permission of Erbil directorate of health. The non-response rate was almost negligible, as few women refused to participate.

The questionnaires were translated by the researcher from English to Arabic language (the mother tongue of the researcher), knowing that the researcher had studied medicine (under-graduate and post-graduate studies) in English language. These forms were put inside envelopes, distributed by women of the *Assyrian Women Union* to ladies attending the Mass (in three churches of Ankawa). Those ladies were informed that the gathered information is confidential in addition to the fact that they contain no space for writing the names. The ladies were asked to fill the forms, put them inside the envelops, seal the envelops and bring them back on the next Mass (next Sunday). It is worth to mention that the above mentioned Women Union is a non-governmental organization (NGO) that was established in 1992 in Erbil, and had a branch in Duhok governorate, Kurdistan region Iraq. After 2003, a branch was established in Baghdad. One of the main tasks of this NGO is women empowerment, and acquainting women with their rights, and with the legislations that respects and documents women rights. Around 2000 women are members of this NGO.

The forms were also translated to Kurdish language by two MSc holders (PhD candidates) whom mother tongue is Kurdish, and their basic and post-graduate medical education were in English. These forms were distributed (in a sealed envelope) to women accompanying patients admitted to the Maternity Teaching Hospital in Erbil city (G2). This hospital is the only public maternity hospital in Erbil city. Women of the *Assyrian Women Union* distributed the questionnaire and then collected the sealed envelopes, and helped women who have problems in understanding the questions, or who were illiterate.

Adjustment for the level of education of women of both groups was done. After analyzing the data of G1, women were categorized into four levels of education according to years of formal education (Table 1). Accordingly, the same number of women lying in each educational level was taken from G2.

**Table 1:** Distribution of sample by educational level

**Years of formal education**	**Group**				**Total**	
	**Ankawa**		**Erbil**			
	**No.**	**%**	**No.**	**%**	**No.**	**%**
<6	2	0.8	2	0.8	4	0.8
6-9	42	16.8	42	16.8	84	16.8
10-12	80	32	80	32	160	32
> 12	126	50.4	126	50.4	252	50.4
**Total**	**250**	**100**	**250**	**100**	**500**	**100**

Data were analyzed using the statistical package for social sciences (SPSS, version 18). Chi square test of association was used to test whether the differences in the prevalence of the three types of violence among the study groups were significant or not. This test was also used to show the association between each of the three types of violence and different factors. Factors found (by bivariate analysis using Chi square test) to be significantly associated with these types of violence, were entered into a binary logistic regression model. The factor *group* (Ankawa/Erbil) was entered into the regression model (of the physical, sexual, and overall violence) in spite of the results of the Chi square test which showed no significant association with the above mentioned types of violence. This variable was entered into the model owing to its importance, as it is part of the research question.

A “P” value of equal or less than 0.05 was considered as statistically significant.

## Results

Table 1 shows that both study groups were comparable (by the intention of the researcher) regarding educational level. Half (50.4%) of the sample were graduates of institutes, colleges, or higher levels (> 12 years of formal education). Only 4 women (0.8%) were illiterate.

Results showed that the overall prevalence of violence in Erbil group (52/250; *20.8%*), was higher than the overall prevalence (47/250; *18.8%*) of Ankawa group (p = 0.575).

Table 2 shows that the prevalence of psychological violence was 40% in Erbil, which was significantly higher than the prevalence (24.8%) of Ankawa (p < 0.001). The rates of physical and sexual violence were also higher in Erbil than Ankawa although the differences were not significant (p = 0.63, p = 0.28 respectively). The prevalence rates of physical and sexual violence in Ankawa were 16.8% and 8% respectively.

**Table 2:** Prevalence of violence by type and residency

	**Group**				**Total**		**P value**
**Type of violence**	**Ankawa**		**Erbil**				
	**N = 250**		**N = 250**		**N = 500**		
	**No.**	**%**	**No.**	**%**	**No.**	**%**	
**Psychological**							
Present	62	24.8	100	40	162	32.4	< 0.001
Absent	188	75.2	150	60	338	67.6	
**Physical**							
Present	42	16.8	46	18.4	88	17.6	0.639
Absent	208	83.2	204	81.6	412	82.4	
**Sexual**							
Present	20	8	27	10.8	47	9.4	0.283
Absent	230	92	223	89.2	453	90.6	

When asking women whether husbands have the right to hit their wives, 26.4% of women in Erbil group vs. 1.4% of women in Ankawa group agreed that men have the right to hit their wives (p < 0.001).

Table 3 shows that the factors found to be associated with physical violence were residing in Erbil compared to Ankawa (OR = 2.7), alcohol drinking by husband (OR = 2.8), woman working as manual worker (OR = 10.6), in addition to marriage without getting the agreement of the woman (OR = 2.2).

**Table 3:** Binary logistic regression analysis of prevalence of physical violence with several covariates

**Factors**	**P**	**OR**	**95% C.I. for OR**	
			**Lower**	**Upper**
**Group (Erbil)**	**.009**	2.711	1.287	5.707
Ankawa (reference)		1.000		
**Smoking status**				
Ex-smoker (reference)		1.000		
Non-smoker	.358	2.751	.318	23.756
Smoker	.998	.000	.000	.
**Alcohol drinking by husband**	**.006**	2.869	1.362	6.043
**Wife employment**				
High professionals (reference)		1.000		
Non-manual skilled	.942	1.030	.462	2.297
Manual (skilled or unskilled)	**.016**	10.640	1.563	72.435
Unemployed	.329	1.556	.641	3.777
**Husband employment**				
High professionals (reference)		1.000		
Non-manual skilled	.089	1.932	.905	4.126
Manual (skilled or unskilled)	.750	.869	.366	2.062
Unemployed	.344	1.718	.560	5.271
**Having children**	.061	4.243	.936	19.235
**House_not owned**	.052	1.691	.994	2.877
**Marriage without woman agreement**	**.048**	2.217	1.007	4.882
**Illegitimate relations of husband**	.097			
None (reference)		1.000		
Present	.077	2.642	.900	7.753
Unknown (by wife)	.137	2.126	.786	5.748
Constant	.000	.003		

In Table 4, only two factors (linked with low socio-economic status) found to be associated with sexual violence and these were women working as manual workers (OR = 43.6), and having no car (OR = 2.3).

**Table 4:** Binary logistic regression analysis of prevalence of sexual violence with several covariates

**Factors**	**P**	**OR**	**95% C.I. for OR**	
			**Lower**	**Upper**
**Group (Erbil)**	.084	1.942	.915	4.123
Ankawa (reference)		1.000		
**Age of marriage (< 18 year)**	.596	1.312	.481	3.575
**Wife employment**				
High professionals (reference)		1.000		
Non-manual skilled	.095	5.876	.734	47.049
Manual (skilled or unskilled)	**.003**	43.687	3.602	529.817
Unemployed	.103	6.052	.694	52.780
**Husband employment**				
High professionals (reference)		1.000		
Non-manual skilled	.160	2.330	.716	7.584
Manual (skilled or unskilled)	.488	1.546	.452	5.290
Unemployed	.380	.347	.033	3.679
**House not owned**	.828	.923	.447	1.904
**Not having car**	**.029**	2.331	1.092	4.977
**Marriage without woman agreement**	.402	1.517	.572	4.018
**Illegitimate relations of husband**	.137			
None (reference)		1.000		
Present	.061	3.129	.947	10.342
Unknown (by wife)	.638	.707	.167	2.989
Constant	.000	.005		

Table 5 shows that the factors significantly associated with psychological violence were residency in Erbil compared to Ankawa, husband employment as a manual worker compared to professional jobs (inverse relation), having children, marriage without woman agreement, and husbands’ illegitimate relations with other women. Odds ratios were 3.6, 0.38, 5.9, 2.9, and 6.3 respectively. Even when the illegitimate relations with other women were unknown by the wives, the table shows that they were more liable for violence (OR = 10.7).

**Table 5:** Binary logistic regression analysis of prevalence of psychological violence with several covariates

**Factors**	**P**	**OR**	**95% C.I. for OR**	
			**Lower**	**Upper**
**Group (Erbil)**	**< 0.001**	3.612	2.200	5.931
Ankawa (reference)		1.000		
**Age of marriage (< 18 years)**	.209	.588	.257	1.347
**Wife employment**				
High professionals (reference)		1.000		
Non-manual skilled	.416	1.318	.677	2.568
Manual (skilled or unskilled)	.693	1.429	.242	8.421
Unemployed	.342	1.632	.594	4.483
**Husband employment**				
High professionals (reference)		1.000		
Non-manual skilled	.153	1.543	.851	2.797
Manual (skilled or unskilled)	**.007**	.380	.188	.769
Unemployed	.679	.810	.298	2.200
**Source of family income**				
Husband (reference)		1.000		
Wife	.211	2.156	.647	7.182
Husband & wife	.100	.514	.233	1.137
**Having children**	**.002**	5.984	1.936	18.492
**Marriage without woman agreement**	**.005**	2.997	1.394	6.444
**Illegitimate relations of husband**				
None (reference)		1.000		
Present	**.001**	6.390	2.053	19.885
Unknown (by wife)	**< 0.001**	10.757	3.840	30.137
Constant	.000	.038		

Factors found to be significantly associated (by bivariate analysis using Chi square test) with overall violence were entered into a binary logistic regression model (Table 6). Results showed that the factors associated with overall violence were: women of G2 (OR = 2.6), women of alcoholic husbands (OR = 2.8), women working as manual workers (OR = 10.4), and women having children (OR = 4.9).

**Table 6:** Binary logistic regression analysis of overall prevalence of violence (physical and/or sexual) with several covariates

**Factors**	**P**	**OR**	**95% CI of OR**	
			**Lower**	**Upper**
**Group (Erbil)**	**.007**	2.619	1.299	5.282
Ankawa (reference)		1.000		
**Smoking woman**	.998	1.222E9	.000	
**Alcoholic husband**	**.004**	2.784	1.384	5.600
**wife employment**	.022			
High professionals (reference)		1.000		
Non-manual skilled	.681	1.178	.539	2.575
Manual (skilled or unskilled)	**.010**	10.444	1.733	62.944
Unemployed	.090	2.077	.891	4.839
**husband employment**	.270			
High professionals (reference)		1.000		
Non-manual skilled	.075	1.934	.935	4.004
Manual (skilled or unskilled)	.580	1.251	.566	2.768
Unemployed	.609	1.333	.443	4.009
**Having children**	**.035**	4.902	1.115	21.546
**Marriage without woman agreement**	.130	1.784	.844	3.773
**Illegitimate relations of husband**	.355			
None (reference)		1.000		
Present	.236	1.874	.662	5.304
Unknown (by wife)	.335	1.595	.618	4.115
Constant	.997	.000		

## Discussion

Results of a previous study done in Erbil at 2010–2011 showed that higher level of education of women (> 12 years of formal education) is significantly associated with lower levels of violence [6]. Other studies showed the same effect [7,8]. So in order to adjust for the effect of educational level as an important predictor of violence against women, the author decided to include the same number of women in each of the educational level categories of the two study groups. Different ethnic groups are residing in Ankawa sub-district, like the Assyrians, the Chaldians, and the Arabs, but all those included were Christians. They share in general the same cultural characteristics and behaviors that are different from the cultural characteristics of people residing in Erbil city, who are Kurdish.

The overall prevalence of violence in Ankawa was 18.8%, and in Erbil was 20.8%. Up to researcher’s knowledge, no previous study had been carried out in Ankawa to assess violence levels among women; even in Erbil there was only one recent study that assessed the past year overall prevalence which was 45.3%. This high rate is attributed to the inclusion of the psychological violence in the overall prevalence in addition to physical and sexual violence. [6] A study done in Baghdad by Abdul Jabbar showed a prevalence of 44% [5], and another one done in Sudan, found that the prevalence among women attending Arda medical center, Omdurman was 41.6% [9]. Other studies showed different results according to the sample and country. The prevalence, 58.7%, was detected in a sample of women attending primary health care centers in Madina, Saudi Arabia [10]. Other studies done in China and Esfahan showed lower results (26% and 29.3% respectively) [11,12].

The study revealed a lower rate of physical violence (16.8%) in Ankawa than in Erbil (18.4%). These rates are little bit more than the rate (15.1%) of the 2010–2011 study done in Erbil [6]. A study done in Iran [13], and another one in Egypt [14] showed rates of 15% and 13% respectively. Lower rates were found in Japan (3%), in New Zealand (5%), in Philippines (6%) and in Thailand (8%), whereas higher rates found in Ethiopia (29%), and in West Bank and Gaza Strip (52%) [15].

Rates of sexual violence of this study (8% in Ankawa, and 10.8% in Erbil) were lower than what was recently reported (12.1%) in Erbil [6]. Nearly comparable rates were found by other studies: 12% in China [11], 11.5% in Samoa, and 12.8% in United Republic of Tanzania city [2]. Higher rates were found in Ethiopia province (44.4%), Bangladesh province (24.2%), Bangladesh city (20.2%), and in United Republic of Tanzania province (18.3%) [2].

The differences in the results of the above mentioned studies could be related to the sampling methods used, and to the characteristics of women included in these studies; and of course related to the country where the study was carried out.

Considering all these factors, it seems that violence against women by intimate partners is a public health problem, not only in Erbil, Iraq, but worldwide. Rates of different types of violence were high in both study groups, although it was higher in Erbil than in Ankawa sub-district. Logistic regression analysis showed that women living in Erbil were more exposed for violence than women living in Ankawa (except for sexual violence). This could be linked to the cultural differences in the two areas, in addition to the differences of the religion of the two groups. The role of religion was investigated in a study done in Nigeria which showed that about 74% of Muslim women supported wife beating compared with a lower proportion (51.5%) among their Christian counterparts [16]. A study done in Ethiopia showed that Muslims were about two times more likely to experience physical violence during lifetime than the Christian women. The same study showed that physical violence during the past 12 months was higher among Muslims and Catholic religion follower than those Orthodox Christian followers [17]. Many verses in Quran are misinterpreted in order to justify men’s superiority. This misinterpretation of religious beliefs together with socio-cultural norms put women in subordinate status and increase violence against them [18].

Results of two studies done in Erbil showed that around 40% of women believed that husbands are justified to beat their wives [6,19], while results of the current study showed a very big difference between the two study groups where 26.4% of women in Erbil group think that men are justified to hit their wives compared with only 1.4% of women in Ankawa group. This may partly explain the cause of the differences in the violence rates among the two study groups.

Results of the present study showed that many factors are associated with violence. One of these factors is wife working as a manual worker (or unemployed), which may reflect poverty and a low socio-economic status. Poverty increases home conflicts and reduces the woman’s power. It also reduces the ability of men to live in a manner that they regard successful [20]. Ellsberg et al. [21] in Nicaragua, Su-Fang et al. [22] in China, and Malco et al. [23] in USA found the positive association between past year intimate partner violence and low socio-economic status.

Having children was associated with higher rates of violence. This could be explained by the pressure imposed upon fathers that is related to their children, which could be a financial one. The more the number of children, the more the economic burden on the family, so the rates of violence are expected to increase. This is consistent with the results of many studies done in Erbil [6], Mexico [24], and Egypt [25].

Husband’s alcohol drinking was associated with physical violence (OR = 2.8) which is an expected finding. Many studies revealed an association between alcohol drinking and violence, such as studies done in China [26], Baghdad-Iraq [5], United Kingdom [27], and Canada [28].

Results showed that illegitimate relations of husbands are associated with violence. Again it is a logical and expected finding, as illegal relationships will lead to poor communication, hence more violence [29]. It coincides with results of other studies done in Baghdad-Iraq [5], and China [11].

The author believe that the sampling method (being non-random, using two different strategies) was the main limitation of the study, but from the other hand, recruiting women from the maternity hospital (the only public maternity hospital in Erbil) will make them feel free to answer what they want to answer and not to be affected by their partners. The churches were selected in Ankawa to be the study setting because it is a place of gathering a large number of Christian women. Conducting a household survey (in order to recruit a random sample) is money and time consuming, and if this survey done in the morning, there would be no chance to meet the working women, and if done in the evening, husbands may be available at home and may interfere with the process of filling the forms or may ask their wives not to participate in the study. In order to adjust for the incomparability between the two study groups (mentioned above), the author have adjusted for the effect of education. Other factors have been adjusted for, using the regression analysis.

## Conclusions

It can be concluded that the prevalence of intimate partner violence against women is still high in both the study settings, although it was higher in Erbil than in Ankawa. The role of culture and religion found to be an important predictor of violence against women. This calls for more efforts by the religious men, and the non-governmental organizations in order to empower women, and let them know their rights. Conducting qualitative research in the future (targeting men, women, and religious people) may provide in depth understanding of the role of religion and culture on violence. Definitely, these acts are worthless without issuing appropriate legislations.

## Competing interests

The author declare that he has no competing interest.

## Author’s contribution

Being a single author, he was responsible for everything in the study, including supervision of the process of data collection.

## Pre-publication history

The pre-publication history for this paper can be accessed here:

http://www.biomedcentral.com/1471-2458/12/800/prepub

## References

[B1] WHO Violence against women. WHO Fact sheet No.239 2009 http://www.who.int/mediacentre/factsheets/fs239/en/

[B2] Garcia-moreno C Jansen HA Ellsberg M Heise L Watts C Prevalence of intimate partner violence: finding from WHO multi-country study on women’s health and domestic violence Lancet 2006 368 1260 1269 10.1016/S0140-6736(06)69523-8 17027732

[B3] Campbell J Jones AS Dienemann J Schollenberger J O’Campo P Gielen AC Intimate partner violence and physical health consequences Arch Intern Med 2002 162 1157 1163 10.1001/archinte.162.10.1157 12020187

[B4] Heise L Ellsberg M Gottemoeller M Ending violence against women Population Reports, Series L, No.11 1999 MD: Johns Hopkins University, School of Public Health, Population Information Program, Baltimore

[B5] Abdul Jabbar MA The prevalence of violence among a group of married women attending two teaching hospitals in Baghdad. Board dissertation 2006 Iraq: Iraqi Commission for Medical Specializations

[B6] Muhammed-Taher HH Prevalence and factors associated with violence among a group of married women in Erbil 2011 Iraq: MSc thesis. Hawler Medical University, College of Medicine

[B7] Odujin O Wife battering in Nigeria Inter J Gynecol Obstet 1993 41 2 159 164 10.1016/0020-7292(93)90699-W 8099032

[B8] Okemgbo CN Omideyi AK Odimegwu CO Prevalence, patterns and correlates of domestic violence in selected Igbo communities of Imo state, Nigeria Afr J Reprod Health 2002 6 2 101 114 10.2307/3583136 12476722

[B9] Ahmed AM Elmardi AE A study of domestic violence among women attending a medical center in Sudan East Mediterr Health J 2005 11 1/2 164 174 16532685

[B10] Tashkandi AA Rasheed P Wife abuse: a hidden problem. A study among Saudi women attending PHC centers East Mediterr Health J 2009 15 5 1242 1253 20214138

[B11] Xu X Zhu F O’Campo P Koinig MA Mock V Campbell J Prevalence of and risk factors for intimate partner violence in China Am J Public Health 2005 95 1 78 85 10.2105/AJPH.2003.023978 15623864 PMC1449856

[B12] Mousavi SM Eshagian A Wife abuse in Esfahan, Islamic Republic of Iran 2002 East Mediterr Health J 2005 11 5/6 860 869 16761655

[B13] Ghazizadeh A Domestic violence: a cross-sectional study in an Iranian city East Mediterr Health J 2005 11 5/6 880 887 16761657

[B14] El-Zanaty F Hussein EM Shawky GA Way AA Kishor S Egypt demographic and health survey 1995 1996 Calverton, Maryland: Macro-International

[B15] Ellsberg M Heise L Researching violence against women: A practical guide for researchers and activities 2005 Washington, DC, United States: WHO, PATH

[B16] Oyediran KA Isiugo-Abanihe UC Perception of Nigerian women on domestic violence: evidence from 2003 Nigeria Demographic and Health Survey Afr J Reprod Heal 2005 9 2 38 53 10.2307/3583461 16485585

[B17] Feseha G G/Mariam A Gerbaba M Intimate partner physical violence among women in Shimelba refugee camp, northern Ethiopia BMC Publ Health 2012 12 125 http://www.biomedcentral.com/1471-2458/12/125 10.1186/1471-2458-12-125 PMC3293014 22340756

[B18] Rabbani F Quershi F Rizvi N Perspective on domestic violence: case study from Karachi, Pakistan East Mediterr Health J 2008 14 2 415 426 18561735

[B19] UNICEF, COSIT, KRSO, MOH Iraq multiple indicator cluster survey, Volume I 2007 Final report Iraq: UNICEF 62

[B20] Jewkes R Intimate partner violence: causes and prevention Lancet 2002 359 1423 1429 10.1016/S0140-6736(02)08357-5 11978358

[B21] Ellsberg M Caldera T Herrera A Winkvist A Kullgren G Domestic violence and emotional distress among Nicaraguan women: results from population-based study Am Psychol 1999 54 30 36

[B22] Guo SF Wu JL Qu CY Yan RY Domestic abuse on women in China before, during, and after pregnancy Chin Med J 2004 117 3 331 336 15043768

[B23] Malcoe LH Duran BM Montgomery JM Socioeconomic disparities in itimate partner violence against native American women: a cross-sectional study BMC med 2004 2 20 http://www.ncbi.nlm.nih.gov/bmc/articles/PMC446227//tool=pubmed 10.1186/1741-7015-2-20 15157273 PMC446227

[B24] Diaz-Olavarrieta C Ellertson C Paz F de Leon SP Alarcon-Segovia D Prevalence of battering among 1780 outpatients at an internal medicine institution in Mexico Soc Sci Med 2002 55 9 1589 1602 10.1016/S0277-9536(01)00293-3 12297245

[B25] Akmatov MK Mikolajczyk RT Labeb S Dhaher E Khann MM Factors associated with wife beating in Egypt: Analysis of two surveys (1995 and 2005) BMC Women’s Health 2008 8 15 http://www.biomedcentral.com/1472-6874/8/15 10.1186/1472-6874-8-15 18801155 PMC2556994

[B26] Parish WL Wang T Laumann EO Pan S Luo Y Intimate partner violence in China: National prevalence, risk factors and associated health problems Int Fam Plan Perspect 2004 30 4 174 181 10.1363/3017404 15590383

[B27] Gilchrist E Johnson R Takriti R Weston S Beech A Kebbell M Domestic violence offenders: characteristics and offending related needs. Findings 217 2003 London: Home office. Research, development, and statistics directorate http://www.homeoffice.gov.uk/rds/pdfs2/r217.pdf

[B28] Krug EG Dahlberg LL Mercy JA Zwi AB Lozano R World report on violence and health 2002 Geneva: WHO

[B29] Brownridge DA Halli SS Living in a sin and sinful living: Toward filling a gap in the explanation of violence against women Aggress Behav 2000 5 6 565 583 10.1016/S1359-1789(99)00003-8

